# Differences Between Mothers and Fathers of Young Children in Their Use of the Internet to Support Healthy Family Lifestyle Behaviors: Cross-Sectional Study

**DOI:** 10.2196/11454

**Published:** 2019-01-23

**Authors:** Rachel Laws, Adam D Walsh, Kylie D Hesketh, Katherine L Downing, Konsita Kuswara, Karen J Campbell

**Affiliations:** 1 Institute for Physical Activity and Nutrition School of Exercise and Nutrition Sciences Deakin University Geelong Australia; 2 Centre for Research Excellence in the Early Prevention of Obesity in Childhood Sydney Australia

**Keywords:** child, family, healthy lifestyle, infant, internet, obesity, parents

## Abstract

**Background:**

In early life, both mothers and fathers are important influences on their children’s diet, active play, and obesity risk. Parents are increasingly relying on the internet and social media as a source of information on all aspects of parenting. However, little is known about the use of Web-based sources of information relevant to family lifestyle behaviors and, in particular, differences between mothers’ and fathers’ use and sociodemographic predictors.

**Objective:**

The objective of this study was to examine if mothers and fathers differ in their use of the internet for information on their own health and their child’s health, feeding, and playing and to examine sociodemographic predictors of the use of the internet for information on these topics.

**Methods:**

We conducted a secondary analysis on data collected from mothers (n=297) and fathers (n=207) participating in the extended Infant Feeding, Activity and Nutrition Trial (InFANT Extend) when their children were 36 months of age. The main outcome variables were the use of the internet for information gathering for parents’ own health and child health, feeding, and playing. Binary logistic regression was used to examine the sociodemographic predictors of outcomes.

**Results:**

Compared with fathers (n=296), a higher proportion of mothers (n=198) used the internet for information on their own health (230, 78.5% vs 93, 46.5%), child health (226, 77.1% vs 84, 42.4%), child feeding (136, 46.3% vs 35, 17.5%), and child play (123, 42.1% vs 28, 14.0%) and intended to use Facebook to connect with other parents (200, 74.9% vs 43, 30.5%). Despite the high use of the internet to support family health behaviors, only 15.9% (47/296) of mothers reported consulting health practitioners for advice and help for their own or their child’s weight, diet, or physical activity. Sociodemographic predictors of internet use differed between mothers and fathers and explained only a small proportion of the variance in internet use to support healthy family lifestyle behaviors.

**Conclusions:**

Our findings support the use of the internet and Facebook as an important potential avenue for reaching mothers with information relevant to their own health, child health, child diet, and active play. However, further research is required to understand the best avenues for engaging fathers with information on healthy family lifestyle behaviors to support this important role in their child’s life.

**Trial Registration:**

ISRCTN Registry ISRCTN81847050; http://www.isrctn.com/ISRCTN81847050

## Introduction

The prevention of childhood obesity remains a significant global public health challenge, with an estimated 41 million children under 5 years of age being overweight or obese [1]. In Australia, nearly one-quarter of 2-4 year-olds are overweight or obese [2], with important health and economic consequences [3] underscoring the importance of early prevention. Lifestyle risk factors for overweight and obesity in early life include a short breastfeeding duration [4], early introduction of solids [4], poor child diet quality [5], short sleep duration [5] as well as low levels of child physical activity [5] and high levels of sedentary behaviors [5], such as screen time exposure. Parents play a primary role in shaping these behaviors through parental modeling, parenting styles, and food, physical activity, and sedentary environments provided [6].

Although much of the existing research has focused on the maternal influences on children’s lifestyle behaviors [7], recent research has also found fathers to have an important influence. Associations between the dietary intakes of fathers and their children have been identified from as early as 20 months of age [8], and these continue through early childhood [9]. Furthermore, parenting styles of fathers [10] as well as feeding practices [11] have been found to be associated with child adiposity [10]. This highlights the importance of considering both maternal and paternal influences on child obesity risk behaviors.

Providing parents with high-quality information and support to inform healthy family lifestyle behaviors is critical. Evidence suggests that parents are actively seeking support in their parenting role. On average, parents in Australia make 11 visits to general practitioners and 14 visits to Maternal and Child Health nurses in the first year of their child’s life, with the majority of these visits unrelated to illness [12]. In addition to health professionals, parents are increasingly relying on the internet and social media for information and support on all aspects of parenting, including family health [13]. A Google survey in 2014 revealed that new and expecting parents undertake nearly 3 times the number of Web-based searches as nonparents [14]. In the United States, 3-quarters of parents use social media, both for information and social support [15]. In line with this, a nationally representative survey conducted in Australia in 2016 revealed that more than 60% of parents and 73% of those with children under 5 years of age used websites, blogs, and Web-based forums to get child health information in the last 6 months. Interestingly, 30% reported that they did not trust this information source [16].

Research on the use of the internet to support lifestyle behaviors important for child obesity prevention is growing but still limited. The use of the internet as the main source of information on nutrition in the general population has grown rapidly in the last decade [17], and research supports that this is also the case among parents of young children [18,19], including those at risk of obesity [20]. There is much less information on the use of the internet by parents to seek information on active childhood play and limiting sedentary behavior. Despite this, excessive screen time and insufficient physical activity rated the top and third top concern of Australian parents in a national health poll [21]. Just 1 Spanish study addresses this, showing that around 30% of parents of young children search for Web-based information on play activities [22]. Our own qualitative research [23] suggests that parents would welcome Web-based sources of support in this area.

Despite the important influence of fathers on child health behaviors [9,24,25], research on the use of the internet and social media to support healthy family lifestyle behaviors has almost exclusively been with mothers, with very few studies [22,26] focusing on fathers. Research suggests that fathers engage in Web-based activities significantly more for general rather than for parenting purposes [27]. In line with this, 1 study of parents of young children in Spain [22] has found that mothers were more likely to search for digital information on child development and family health compared to fathers, but there were no differences in seeking information on play activities. Another study [26] found no significant difference in the use of the internet for health information between expectant mothers and fathers; however, the sample of fathers was small (n=21), limiting the ability to draw robust conclusions. In terms of social media, Facebook was found to be the most popular among parents in the United States; however, it was used more by mothers (81%) compared with fathers (66%) for information and social support [15]. Further research is required to examine fathers’ use of the internet to support healthy family lifestyle behaviors.

We also know little about how the use of the internet for family health information seeking varies according to parents’ socioeconomic position, ethnicity, or age. There is a debate in the general parenting literature about the existence of a digital divide in the use of the internet and Web-based sources of health information according to parents’ sociodemographic characteristics. Although internet access is near universal, some research suggests that parents of lower socioeconomic position (as indicated by education, income, or the use of services for vulnerable parents) are less likely to use the internet for health information seeking [22,28,29] but equally likely to use social media for these purposes [28]. In line with this, Guerra-Reyes and colleagues [30] reported that in postpartum women, college graduates searched authoritative Web-based sources, while nongraduates preferred forums. Other studies, however, have found no difference in the use of the internet for information on pregnancy [26] and personal or child health information according to parents’ education levels [20]. Again, these studies were predominantly among mothers, with very few studies specifically examining sociodemographic predictors of fathers’ use of the internet for family health information seeking.

The aim of this paper is to examine if mothers and fathers differ in the use of the internet for information on their own health and their child’s health, feeding, and playing and to examine sociodemographic predictors of the use of the internet for information on these topics. This will provide new insights into the differing patterns of internet use by mothers and fathers to support healthy family lifestyle behaviors, which is important in informing the future development of targeted interventions in this area.

## Methods

### Study Context and Participant Recruitment

This study undertook a secondary analysis of data collected as part of the extended Infant Feeding, Activity and Nutrition Trial (InFANT Extend) Program: a cluster randomized controlled trial of a 33-month parent-focused child obesity prevention intervention. Details of the trial and participant recruitment have been reported elsewhere [31]. In brief, the trial aimed to test the effectiveness of a 6-session group-based program delivered to first-time parents when infants were 3-18 months of age, followed by quarterly newsletters from 18-36 months of age. Control participants received usual care. Participants were recruited from first-time parent groups within 7 purposively selected socioeconomically disadvantaged local government areas in Melbourne, Australia. Individual parents were eligible to participate if they gave informed written consent, were first to time parents, and were literate in English. Both the main caregiver and partner were invited to participate in the program and data collection. Infants with chronic health problems likely to influence height, weight, levels of physical activity, or eating habits were excluded from analyses but were permitted to participate in the program.

### Data Collection

This study analyzed data collected from main caregivers and partners participating in the InFANT Extend trial when children were 36 months of age (between 2014-2015). Participants were asked about their use of the internet in general and for information on their own health, dieting, recipes, child health, how to feed their baby, and how to play with their baby. Participants were asked if and how often they used Facebook and whether they could imagine using Facebook to connect with other parents. Sociodemographic variables collected included parent education level, country of birth, relationship status, employment status, income, and self-rated health status. Participants’ perception of the attention they paid to personal health habits and weight control were collected using a 5 point Likert scale from “none” to “very much.” Participants were asked about how well they were coping with life at present from “not at all” to “extremely well.” The main caregiver was also asked about whether they had consulted with a health practitioner for advice and help for their own or child weight, diet, or physical activity; the type of practitioner consulted as well as whether they had access to the internet, a tablet, desktop computer, laptop, or smartphone at home. All data were collected using paper-based surveys mailed to participants with paid reply envelopes.

### Data Analyses

The main outcome variables of interest were the use of the internet for information on parents’ own health and child health, feeding, and playing. Preliminary analyses showed no significant differences in these variables between the intervention and control group participants; hence, data were analyzed for both groups combined. However, the treatment group was controlled for in the analysis; main caregiver and partner data were analyzed separately. All main caregiver surveys were completed by mothers, and all partner surveys were completed by fathers with the exception of 5 surveys (2 mothers, 2 stepfathers, and 1 participant who did not identify their relationship with the child), which were excluded for the purposes of this analysis. Initial analyses were conducted to describe each sample (mothers and fathers) and their use of the internet. Binary logistic regression analyses were used to examine sociodemographic predictors of (1) mothers’ use of the internet for information on child feeding and playing and (2) fathers’ use of the internet for information on their own health and their child’s health. These outcome variables were chosen for logistic regression analysis because they showed the most variability (around 40%-50% of the sample responding positively), whereas other variables had very high positive responses (>75%; ie, mothers’ use of the internet for own health and child) or very low positive responses (<20%; ie, fathers’ use of the internet for information on child feeding and playing), limiting the ability to examine predictors. The selection of sociodemographic and other independent variables to be included in the models was based on existing literature and hypotheses about factors potentially important in influencing internet use. Independent variables showing little variation (ie, relationship status and employment status in fathers showed >90% were married and worked full-time) were considered redundant and not included in the models.

### Ethics

This study was approved by the Deakin University Human Ethics Research Committee.

## Results

### Participant Characteristics

A total of 57.8% (297/514) main caregivers and 47.8% (207/433) partners participating in the trial completed the survey when their child was 36 months of age. There were no significant differences in any baseline characteristics of those who completed the survey at 36 months and those lost to follow-up, with the exception that those lost to follow-up had a higher prepregnancy body mass index (BMI). Participant characteristics are shown in Table 1. A higher proportion of mothers had a university education than fathers. Just under half of mothers were not in paid employment and were looking after children full-time, and a similar proportion worked part-time. Just over 10% (31/297, 10.4%) of mothers worked full-time compared with over 90% (179/197, 90.9%) of fathers. Most parents were married or in a de facto relationship and around 80% (387/487, 79.5%) of all parents were born in Australia. Self-rated health status was high for both mothers and fathers, and just under half rated their attention to personal health habits as high.

**Table 1:** Participant characteristics when their child was aged 3 years.

Characteristics		Mothers (n=297)	Fathers (n=202)
Age in years, mean (SD); range		35.2 (4.2); 22.3-47.9	37.3 (5.4); 26.2-57.8
**Education, n (%^a^)**			
	High school education or less	36 (12.4)	33 (16.8)
	Trade, certificate, or diploma	80 (27.6)	78 (39.8)
	University degree or higher degree	174 (60.0)	85 (43.4)
**Relationship status, n (%^a^)**			
	Married or de facto relationship	274 (92.3)	N/A^b^
	Separated, divorced, or widowed	12 (4.0)	N/A
	Never married	11 (3.7)	N/A
**Employment status, n (%^a^)**			
	Working full-time	31 (10.4)	179 (90.9)
	Working part-time	132 (44.4)	9 (4.6)
	Unemployed or laid off	1 (0.3)	4 (2.0)
	Keeping house full-time	130(43.8)	2 (1.0)
	Studying full-time	3 (1.0)	3 (1.5)
Health care card, n (%^a^)		35 (11.8)	N/A
**Country of birth, n (%^a^)**			
	Australia	229 (78.7)	158 (80.6)
	Other	62 (21.3)	38 (19.4)
**Self-rated health status, n (%^a^)**			
	Excellent	19 (6.4)	20 (9.9)
	Very good or good	226 (76.1)	156 (77.2)
	Fair	49 (16.5)	24 (11.9)
	Poor	3 (1.0)	2 (1.0)
**Attention to personal health habits, n (%^a^)**			
	None or little	40 (13.5)	32 (15.8)
	Some	121 (40.7)	78 (38.6)
	Much or very much	136 (45.8)	92 (45.5)
**Attention paid to controlling weight, n (%^a^)**			
	None or little	90 (30.3)	55(27.4)
	Some	115 (38.7)	82 (40.8)
	Much or very much	92 (31.0)	64 (31.8)
**Coping with life at present, n (%^a^)**			
	Not at all or little	19 (6.4)	10 (5.0)
	Fairly well	135 (45.5)	87 (43.1)
	Very or extremely well	143 (48.1)	105 (52.0)

^a^Percentages relate to samples with valid data for each variable; missing data were excluded.

^b^N/A: not applicable (as these questions were only asked of mothers).

### Use of the Internet to Support Healthy Family Lifestyle Behaviors

Among the 297 mothers, 292 (98.3%) reported having internet access at home and 256 (86.1%) had a tablet, 292 (98.3%) a smartphone, 254 (85.5%) a laptop, and 149 (50.2%) a desktop computer. Although almost all mothers and fathers reported using the internet, there were distinct differences in use of the internet for health and lifestyle-related information between mothers and fathers (Table 2). Over 90% of mothers reported using the internet for recipes and around 3-quarters for information on their own health and their child’s health compared with less than half of fathers using the internet for their own health or their child’s health. Around half of the mothers reported using the internet for information on child feeding and a similar proportion for play compared with only 17.5% (35/200) and 14.0% (28/200) of fathers, respectively. Around 90% (266/297, 89.9%) of mothers and 70% (140/198, 69.7%) of fathers reported being a member of Facebook, with frequent use in both groups. However, less than a third of fathers reported that they could imagine using Facebook to connect with other parents, compared with nearly 3-quarters of mothers.

### Health Professional Consultations for Diet, Activity, and Weight

In contrast to the high proportion of mothers reporting using the internet for information on dieting, child feeding, and playing, only 15.9% (47/296) reported seeking health professional advice and help for their own or their child’s weight, diet, or physical activity (Table 3); this question was only asked of primary caregivers. General practitioners (family doctors) and pediatricians were the most common health professionals consulted.

**Table 2:** Internet and Facebook use by mothers and fathers.

Use		Mothers (n=297), n (%^a^)	Fathers (n=202), n (%^a^)
Use the internet		296 (99.7)	198 (98.5)
**Use the internet to access information on**			
	Your health	230 (78.5)	93 (46.5)
	Dieting or your diet	109 (37.2)	59 (29.6)
	Fitness	N/A^b,c^	81 (40.5)
	Child health	226 (77.1)	84 (42.4)
	How to feed your child	136 (46.3)	35 (17.5)
	How to play with your child	123 (42.1)	28 (14.0)
	Recipes	274 (92.9)	N/A^d^
Member of Facebook		266 (89.9)	140 (69.7)
**Frequency of Facebook use**			
	Once a week or less	24 (9.4)	18 (12.9)
	A few times a week	17 (6.4)	17 (12.2)
	Once a day	57 (21.3)	38 (27.3)
	Several times a day	168 (62.9)	66 (47.5)
**Could you imagine using Facebook to connect with other parents**			
	Yes	200 (74.9)	43 (30.5)
	No	19 (7.1)	65 (46.1)
	Maybe	48 (18.0)	33 (23.4)

^a^Percentages relate to samples with valid data for each variable; missing data were excluded.

^b^N/A: not applicable.

^c^These questions were only asked of fathers.

^d^These questions were only asked of mothers.

**Table 3:** Mothers’ use of health practitioners for advice and help for own weight or child weight, diet, or physical activity (N=296).

Mothers’ use of health practitioners	Mothers, n (%)
Any health professional advice since birth	47 (15.9)
Maternal and child health telephone line	12 (4.1)
Mother and baby center (day stay)	6 (2.0)
Mother and baby center (overnight stay)	3 (1.0)
Home visit or outreach nurse	5 (1.7)
General practitioner or family doctor	23 (7.8)
Pediatrician	19 (6.4)
Dietitian	14 (4.7)
Chiropractor, naturopath, or osteopath	12 (4.1)
Other health professional	12 (4.1)

### Predictors of Mothers’ Use of the Internet for Information on Child Feeding and Playing

Predictors of mothers’ use of the internet for information on child feeding and playing are shown in Tables 4 and 5 respectively. Nonworking mothers were 1.7 times more likely to seek information from the internet on child feeding compared with mothers who were working or studying, with employment status the only significant predictor in the model. Mothers born outside Australia were also more likely to use the internet for information on child feeding compared with Australian-born mothers; however, this did not reach statistical significance. The model explained 37%-50% of the variance in mothers’ use of the internet for information on child feeding. There were no significant sociodemographic predictors of internet use for information on child play; however, there was a trend for university-educated mothers to be more likely to seek information on child play compared with their less educated counterparts (Table 5). This model explained only 25%-33% of the variance in mothers’ use of the internet for information on child play.

**Table 4:** Predictors of internet use by mothers for information on child feeding when their child was aged 3 years.

Predictor		β (SE β)	Odds ratio (95% CI)	*P* value
Age		0.01 (0.03)	1.01 (0.95-1.07)	.87
**Education**				.54
	Nonuniversity educated	Reference	1.00	
	University educated	−0.16 (0.26)	0.85 (0.51-1.43)	
**Employment status**				.02
	Working or studying	Reference	1.00	
	Not working (keeping house or unemployed)	0.56 (0.25)	1.76 (1.08-2.87)	
**Country of birth**				.07
	Australia	Reference		
	Other	0.56 (0.32)	1.76 (0.95-3.25)	
Child body mass index *z*-score age 3 years		0.02 (0.15)	1.02 (0.76-1.37)	.91
**Attention paid to personal health habits**				.23
	None, little, or some	Reference		
	Much or very much	0.30 (0.25)	1.35 (0.83-2.20)	
**Treatment group**				.15
	Control	Reference		
	Intervention	0.36 (0.25)	1.43 (0.88-2.31)	

**Table 5:** Predictors of internet use by mothers for information on child play when their child was aged 3 years.

Predictor		β (SE β)	Odds ratio (95% CI)	*P* value
Age		−0.03 (0.03)	0.97 (0.91-1.03)	.33
**Education**				.09
	Nonuniversity educated	Reference	1.00	
	University educated	0.45 (0.27)	1.57 (0.93-2.66)	
**Employment status**				.19
	Working or studying	Reference	1.00	
	Not working (keeping house or unemployed)	0.33 (0.25)	1.39 (0.85-2.27)	
**Country of birth**				.87
	Australia	Reference	1.00	
	Other	0.05 (0.31)	1.05 (0.57-1.94)	
Child body mass index *z*-score age 3 years		−0.09 (0.15)	0.92 (0.68-1.24)	.57
**Attention paid to personal health habits**				.54
	None, little, or some	Reference		
	Much or very much	0.16 (0.25)	1.17 (0.72-1.90)	
**Treatment group**				.30
	Control	Reference		
	Intervention	0.26 (0.25)	1.29 (0.80-2.11)	

### Predictors of Fathers’ Use of the Internet for Information on Their Own Health and Their Child’s Health

Fathers’ reported attention to personal health habits was the only significant predictor of the use of the internet for their own health. There was a trend for fathers with higher levels of education to be more likely to use the internet for their own health; however, this did not reach statistical significance (Table 6). This model only explained 8%-11% of the variance in fathers’ use of the internet for information on their own health. A younger age, higher education level, and greater reported attention to personal health habits were all significant predictors of the use of the internet for information on child health (Table 7). Fathers born outside Australia were also more likely to use the internet for child health, but this did not reach statistical significance. This model explained 13%-18% of the variance in fathers’ use of the internet for information on their child’s health.

**Table 6:** Predictors of use of the internet by fathers for information on their own health when their child was aged 3 years.

Predictor		β (SE β)	Odds ratio (95% CI)	*P* value
Age		−0.04 (0.03)	0.96 (0.90-1.02)	.16
**Education**				.08
	Nonuniversity educated	Reference	1.00	
	University educated	0.56 (0.32)	1.74 (0.93-3.27)	
**Country of birth**				.41
	Australia	Reference	1.00	
	Other	0.33 (0.40)	1.39 (0.64-3.00)	
**Attention paid to personal health habits**				
	None or little	Reference	1.00	.02
	Some	−0.01 (4.63)	0.99 (0.40-2.45)	.98
	Much or very much	0.88 (0.45)	2.42 (0.99-5.88)	.05^a^
Fathers’ body mass index		0.04 (0.05)	1.04 (0.95-1.14)	.37
**Treatment group**				.61
	Control	Reference	1.00	
	Intervention	−0.16 (0.31)	0.85 (0.47-1.56)	

**Table 7:** Predictors of use of the internet by fathers for information on their child’s health when their child was aged 3 years.

Predictor		β (SE β)	Odds ratio (95% CI)	*P* value
Age		−0.07 (0.03)	0.93 (0.87-0.99)	.03
**Education**				.001
	Nonuniversity educated	Reference		
	University educated	1.07 (0.34)	2.92 (1.51-5.66)	
**Country of birth**				.08
	Australia	Reference		
	Other	0.71 (0.41)	2.04 (0.91-4.55)	
**Attention paid to personal health habits**				
	None or little	Reference		.05
	Some	0.97 (0.53)	2.62 (0.93-7.41)	.07
	Much or very much	1.26 (0.52)	3.52 (1.28-9.67)	.02
Fathers’ body mass index		0.004 (0.05)	1.004 (0.91-1.11)	.93
**Treatment group**				.93
	Control	Reference		
	Intervention	−0.03 (0.32)	0.97 (0.52-1.83)	

## Discussion

### Principal Findings

This is 1 of few studies to compare mothers’ and fathers’ use of the internet to seek information on their own health and their child’s health, feeding, and playing and to examine sociodemographic predictors of the use of the internet in these areas. Despite the similar use of the internet by both parents, mothers were found to use the internet more than fathers to seek information in all the family health domains examined. Mothers also reported much higher use of the internet for information relevant to childhood obesity prevention compared with consulting health professionals for advice in this area. Sociodemographic predictors varied between mothers and fathers and the specific topics examined, highlighting the nuances in health information seeking behaviors of parents.

A key finding of interest in this study was that although nearly half of the mothers reported using the internet for information on child feeding and playing, less than 1-fifth reported consulting a health professional on these matters. This is in line with previous research that suggests mothers of young children prefer digital media because it provides them with free information when they need it the most and at the times when they have opportunities to access it [32]. In contrast, a lack of time and childcare, inconvenient scheduling, and lack of awareness of services or programs have previously been reported as the top barriers to accessing more traditional face-to-face parenting services and programs [18]. This highlights the importance of Web-based digital media as a potential avenue for the delivery of programs to mothers to promote healthy lifestyle behaviors for the whole family. The findings also underscore the importance of understanding the quality of parenting information accessed by parents on the internet.

Despite the almost universal use of the internet by parents in this study, we found, in line with previous research, a much higher proportion of mothers compared with fathers reported using the internet for family health information seeking [22,27]. These differences may reflect the division of responsibility in child rearing, with women more likely to be responsible for cooking and child feeding and gatekeepers for family health and nutrition [33]. However, our own qualitative research [34] shows that fathers believe that they have shared responsibility with respect to the dietary and physical activity behaviors of their young children but felt frustrated by the lack of useable or, at times, conflicting information. This suggests that difficulties accessing or identifying quality Web-based resources may be a barrier for fathers, particularly given the plethora of information available, with limited time exacerbating this issue. Parenting websites also typically target mothers as the primary audience and, therefore, may cater to their interest at the expense of engaging fathers who have been found to have negative attitudes to Web-based parenting resources [27]. Further research is required to explore the best mode of engaging fathers in Web-based parenting resources targeting child lifestyle behaviors.

Our findings regarding the existence of a digital divide in the use of the internet for healthy lifestyle information differed between mothers and fathers. For mothers, maternal age and education were not significant predictors of the use of the internet for information on child feeding and playing. The only significant predictor was employment status, with nonworking mothers nearly twice as likely to search for information on the internet about feeding their child. This may reflect time available outside of paid employment and the more frequent provision of meals to young children among nonworking mothers. In contrast, our findings do suggest a digital divide exists for fathers, with younger, more health conscious, and university-educated fathers more likely to use the internet for information on child health. Again, further research is required to better understand how to engage fathers in digital information relevant to child health and family lifestyle behaviors.

A novel finding in this study was the influence of ethnicity on Web-based information seeking relevant to family health. There was a trend (nonsignificant) for parents born outside Australia to be more likely than Australian-born parents to seek information on the internet related to child feeding (mothers) or child health (fathers). This is in line with 2 qualitative studies that showed that barriers related to language, cultural beliefs, and unfamiliarity with health services drive immigrants to seek postnatal health information primarily from their personal network and the internet [35,36]. Further research is required to understand how ethnic minority groups seek information relevant to family lifestyle behaviors and to better understand the potential for Web-based resources in reaching these parents.

It is important to point out that the multiple regression models only explained a relatively small proportion of the variance in the use of the internet by parents, ranging from as little as 8% of the variance in fathers’ use of the internet for their own health to explaining up to 50% of variance in mothers’ use of the internet for child feeding information. As is the case with most health behavior research, this suggests that there are many more unmeasured factors influencing the Web-based information seeking behaviors of parents in this study. In support of this, literature in the digital parenting space suggests patterns of health information seeking are complex and reflect differing perceptions of information availability and usefulness, access to health services [37], and comfort with technology [28]. As such, further research is required to unpack the Web-based health information seeking behaviors of parents to maximize the reach and potential use of Web-based parenting resources, particularly in the area of family healthy lifestyle behaviors.

While both mothers and fathers in our study reported being frequent users of Facebook, less than a third of fathers in this study could imagine using Facebook to connect with other parents, compared to nearly 3-quarters of mothers. This may reflect mothers’ preferences to connect with other mothers more generally rather than specific preferences for the use of Facebook for this purpose. However, research [15] does suggest that mothers are more likely than fathers to use social media for parenting information as well as social and emotional support for parenting issues. Social media has been proposed as an important emerging context for social influence on mothers’ child feeding practices and, thus, a potential avenue for delivery of child obesity prevention interventions [38]. Further research is required to better understand how social media influences obesity-related behaviors in families and how effective interventions could be developed using these platforms.

### Strengths and Limitations

This study has a number of strengths and limitations. A key strength of this study was the relatively large sample of parents, enabling predictors of internet use for family health information seeking to be examined separately for mothers and fathers, unique within the existing literature. However, the parents were recruited to participate in a child obesity prevention intervention and, hence, may be more interested in family health behaviors, limiting generalizability. The questions on internet use were not derived from a validated scale or pretested prior to use. As data were collected in 2014-2015, the findings may not reflect parents’ current digital health information seeking practices; in particular, we did not examine the use of other social media beyond Facebook. The findings do, however, provide unique insights and ideas for future research.

### Conclusions

Our findings support the use of the internet and Facebook as important potential avenues for reaching mothers with information relevant to family lifestyle behaviors, given their frequent use and apparent preference for these sources over consulting health professionals. However, fewer fathers reported using the internet for information relevant to family lifestyle behaviors. Further research is required to understand how best to engage fathers in Web-based parenting resources targeting child obesity prevention, particularly the less educated and less health conscious fathers who are the least likely to seek this information but are likely in greater need of support. Our findings also warrant further investigation of the internet as a potential avenue for reaching ethnic minority parents. The findings from this study, however, only explained a proportion of the variance in the health information seeking behaviors of parents, highlighting the importance of further research in this area to uncover the potential of digital parenting resources and other modes of delivery in supporting healthy lifestyle behaviors in families.

## Abbreviations

InFANT: Infant Feeding, Activity and Nutrition Trial
BMI: body mass index
